# Analysis of Clinical Outcome of Patients with Poorly Differentiated Thyroid Carcinoma

**DOI:** 10.5402/2011/308029

**Published:** 2011-03-29

**Authors:** Katsuhiro Tanaka, Hiroshi Sonoo, Wataru Saito, Yusuke Ohta, Toshiro Shimo, Mai Sohda, Yutaka Yamamoto, Junichi Kurebayashi

**Affiliations:** Department of Breast and Thyroid Surgery, Kawasaki Medical School, 577 Matsushima, Kurashiki 701-0192, Japan

## Abstract

*Background*. We retrospectively analyzed whether poor differentiation is the independent prognostic factor for thyroid carcinoma or not. *Methods*. The subjects were 29 patients with PDTC who were treated between April 1996 and March 2006 to compare with those of well-differentiated papillary carcinoma patients (*n* = 227). *Results*. The relapse free (RFS), distant relapse-free survival and cause-specific survival, rates were significantly lower in patients with PDTC (*P* < .0001, *P* < .001, and *P* < .05). After classification into focal (<10%) and diffuse type (over 10%) of PDTC, there were no significant differences in RFS and cause-specific survival due to component type or proportion of poorly differentiated component. On multivariate analysis, poor differentiation (*P* < .0005, RR = 4.456, 95% CI; 1.953–10.167) and extrathyroidal infiltration (*P* < .05, RR = 2.898, 95% CI; 1.278–6.572) showed a significant impact on DFS, and poor differentiation (*P* < .05, RR = 9.343, 1.314–66.453) and age (*P* < .005, RR = 1.306, 1.103–1.547) significantly impacted cause-specific survival. *Conclusion*. Poor differentiation was an independent factor for survival. Distant relapse was significantly more common among PDTC patients, and systemic therapy might be warranted.

## 1. Introduction

Poorly differentiated thyroid carcinoma (PDTC) was first proposed by Sakamoto et al. in 1983 [1] and separately categorized by the 2004 WHO classification of Endocrine Tumors [2]. Based on the Turin proposal in 2006, PDTC demonstrates solid, trabecular, and insular components, the absence of conventional nuclear patterns of papillary carcinoma, and the presence of at least one of the following features: convoluted nuclei, mitotic activity, or necrosis [3]. 

Some reports have shown a worse prognosis for PDTC to be compared with that of well-differentiated thyroid carcinoma (WDTC). Anaplastic thyroid carcinoma is a well-known to be lethal regardless of the proportion of incident anaplastic carcinoma within well-differentiated carcinoma. Even when PDTC is a minor component within the tumor, an aggressive behavior has been reported [4, 5]. However, it has been reported that minor focal distribution of an insular component does not affect the prognosis [6, 7]. PDTCs are divided into focal PDTC (<10%) and diffuse PDTC (>10%) based on the proportion of the poorly differentiated component [2]. Thus, the clinical behaviors of poorly differentiated thyroid carcinoma remain controversial, and it is unclear whether the proportion of the poorly differentiated component affects its prognosis.

We retrospectively analyzed the outcome of patients with poorly differentiated thyroid carcinoma who were initially treated in our hospital between April 1996 and March 2006. Furthermore, we compared findings with those of well-differentiated papillary carcinoma patients who underwent surgery during the same period in order to evaluate the characteristics of the subjects.

## 2. Patients and Methods

This is a cohort study to identify the characteristics of patients with PDTC treated by primary surgery in our hospital between April 1996 and March 2006. Clinicopathological findings were compared with those of WDTC patients who underwent surgery during the same period in order to evaluate the clinical characteristics of these subjects. The initial surgical procedures were ipsilateral hemithyroidectomy for patients with tumors over 1.0 cm in diameter and total lobectomy for those with tumors smaller than 1.0 cm in diameter. Total thyroidectomy was performed for patients with distant metastasis, with tumor invasion of adjacent organs or with tumor confined to bilateral lobes on preoperative examination and intraoperative determination. All our papillary thyroid carcinoma patients undergo modified radical dissection of the cervical lymph nodes (central component only or central and lateral components). We do not routinely administer radioiodine as adjuvant therapy; however, thyrotropin (TSH) suppression therapy was achieved with thyroxine (T4) during the course of the disease as an adjuvant therapy after surgery for all enrolled patients. All the patients were followed by checking serum thyroglobulin value, cervical palpation, and chest film every 6 months postoperatively. The relapse-free and distant relapse-free survivals were defined as survival without any recurrence and without any distant recurrence, respectively. The choice of standard surgical procedures and not useing postoperative routine radioiodine therapy in our hospital are not unique in Japan [8, 9].

PDTC was diagnosed when poorly differentiated subtypes were recognized regardless of the proportion. The ratio of each PDTC component to WDTC was calculated in order to divide the two groups into the focal (<10%) or diffuse (>10%) type. Informed consent was obtained from all enrolled patients. The clinical data have been deidentified under anonymity, and patient information was protected in this retrospective study. The Mann-Whitney *U* test, the Kaplan-Meier method, and Cox's proportional hazard model were used for analyses, and  *P* < .05 was considered significant.

## 3. Results

### 3.1. Clinical Characteristics

Clinical characteristics are shown in Table 1. The patients with PDTC were 29, they were treated during that period (control patients: 227). Of these 29 patients, there were 7 men and 22 women. The median patient age was 57, and mean primary tumor size was 3.1 ± 1.8 cm. The primary tumor in patients with PDTC was significantly larger than that in those with well-differentiated tumor (*P* < .0001). Histological lymph node involvement and extrathyroidal infiltration (EX) were positive in 21 (72.4%) and 16 (55.2%) patients with PDTC, respectively. 

The distribution of three subtypes of poorly differentiated thyroid carcinoma was 27.6% solid type, 17.2% insular type, 24.1% trabecular type, and 31.0% solid and trabecular type. The distribution of focal and diffuse components of PDTC was 37.9% (11 of 29) and 62.1% (18 of 29), respectively.

Table 2 shows the clinical characteristics of the focal and diffuse types of PDTC. There was no difference between focal and diffuse types in age, gender, lymph node involvement, tumor size, or pathological type of PDTC. However, the ratio of extrathyroidal infiltration to an adjacent organ such as the trachea and/or esophagus (EX2) was 57.1% (8 of 14) in the diffuse type and 7.1% (1 of 15) in the focal type. There was a significant difference in the presence of EX2 (*P* < .01).

### 3.2. Outcome

**Figure 1:**
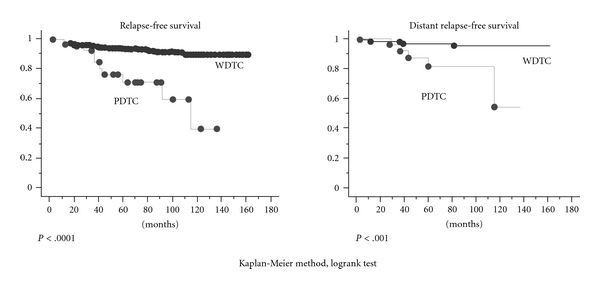
The 10-year relapse-free survival rate was 32.1% in poorly differentiated carcinoma (8.0% in well-differentiated carcinoma, *P* < .05). Using the Kaplan-Meier method, relapse-free and distant relapse-free survival rates were significantly lower among patients with poorly differentiated carcinoma (*P* < .0001 and *P* < .001, resp.).

**Figure 2:**
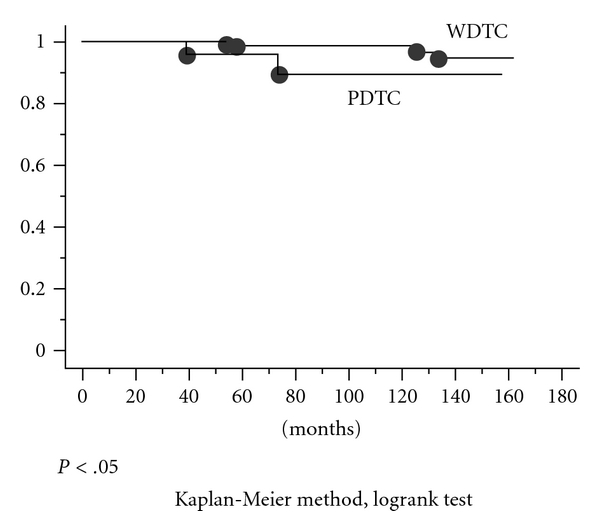
Of 29 patients with poorly differentiated carcinoma, two died of disease, and of 227 with well-differentiated carcinoma, four died of disease. The 10-year cause-specific survival rates were 89.3% for PDTC and 98.7% for WDTC. Despite the low number of disease-related deaths, there was a significant difference between the two groups (*P* < .05).

Median followup periods for PDTC and WDTC patients were 75.9 and 89.3 months, respectively. The 10-year recurrence rate was 32.1% for poorly differentiated carcinoma (8.0% for well-differentiated carcinoma, *P* < .05). Using the Kaplan-Meier method, relapse-free and distant relapse-free survival rates were significantly lower in PDTC (*P* < .0001 and *P* < .001, resp., Figure 1). Of 29 patients with PDTC, two died of disease, and of 227 with WDTC, four died of disease. The 10-year cause-specific survival rates were 89.3% for PDTC and 98.7% for WDTC. Despite the low number of disease-related deaths, there was a significant difference between groups (*P* < .05, Figure 2). In the focal and diffuse type, there were no significant differences in DFS and cause-specific survival. However, patients who died of disease were only found in diffuse type.

Table 3 shows the results of multivariate analysis for DFS and cause-specific survival in all patients (*n* = 256). Analyzed factors were age, gender, tumor size, lymph node involvement, extrathyroidal infiltration, poor differentiation, and population of poor differentiation. Poor differentiation (*P* < .0005, RR = 4.456, 95% CI; 1.953–10.167) and extrathyroidal infiltration (*P* < .05, RR = 2.898, 95% CI; 1.278–6.572) were significant factors for DFS. Furthermore, poor differentiation (*P* < .05, RR = 9.343, 1.314–66.453) and older age (*P* < .005, RR = 1.306, 1.103–1.547) significantly affected cause-specific survival. The proportion of PDTC did not influence DFS and cause-specific survival.

## 4. Discussion

In Japan, the application of surgical methods and use of postoperative radioiodine therapy for WDTC are unique compared with other countries [8, 9]. In our findings, the 10-year cause-specific survival rates were 89.3% for PDTC and 98.7% for WDTC. In other countries, 10-year and 40-year relative survival rate was reported as 93% [10] and 94% [11], respectively. Our data is not inferior to those of other countries. Actually, it is still controversial that total thyroidectomy followed by postoperative radioiodine therapy and TSH suppressive therapy is associated with improved survival for all patients with WDTC [12, 13]. For high-risk patients with WDTC, this standard therapy might be beneficial [12, 13].

Poorly differentiated thyroid carcinoma consists of three pathological subtypes, solid, insular, and trabecular [2]. In 2006, a consensus meeting was held in Turin and PDTC was discussed by experts from many different countries. Volante and Papotti published a summary of the established consensus and remaining controversies [14]. According to the established consensus, solid, trabecular, and insular growth patterns should be predominant [14]. Ito et al. have shown the differences among three definitions proposed by Sakamoto et al. [1], WHO [2], and Turin [12] for PDTC [15]. 

Many reports have demonstrated a poorer survival among poorly differentiated carcinoma patients than among those with well-differentiated carcinoma [4–7, 16]. In our study, patients with PDTC also showed significantly poorer outcome compared to that of those with WDTC. Especially, PDTC patients showed a significantly worse outcome in distant relapse-free survival. On multivariate analysis, poor differentiation was one of the independent prognostic factors for both DFS and cause-specific survival. Each relative risk was 4.456 and 9.343, respectively. Similar to our findings, poor differentiation has previously been demonstrated to be an independent factor for survival [17].

 Thus, poor differentiation is associated with a worse prognosis. However, it remains controversial whether the proportion showing poor differentiation (focal or diffuse) affects the outcome of patients with PDTC. In our study, the distribution of patients showing focal and diffuse components of PDTC was 37.9% (11 of 29) and 62.1% (18 of 29), respectively. Even when PDTC is a minor component of the tumor, an aggressive behavior has been demonstrated [4, 5]. However, it has been reported that a minor focal distribution of the insular component does not affect the prognosis [6, 7]. In our series, there was no significant difference in DFS or cause-specific survival between patients with focal or diffuse PDTC. However, patients with diffuse PDTC showed a significantly higher ratio of extrathyroidal infiltration. This might be an indication of aggressive local disease in diffuse type of PDTC. Despite the low number of disease-related deaths in this series, patients with PDTC showed a significantly rate of disease-related death.

Decreased thyrotropin receptor (TSH-R) has been shown in PDTC compared with that in WDTC [18]. The TSH-R expression level has been reported to correlate with unfavorable clinical features [19]. There are some reports of in vitro studies demonstrating a lower expression of sodium iodide symporter in poorly differentiated thyroid carcinoma cell lines [20, 21]. Thus, the reduction of thyroid differentiation markers such as TSH-R, sodium iodide symporter, and thyroglobulin might mean worse prognosis. Although it remains unclear whether radioiodine therapy is useful in PDTC [8], distant relapse and local invasiveness were significantly more common in PDTC patients. Therefore, for patients with PDTC, systemic treatment might be warranted during the initial therapy as high-risk patients.

## Figures and Tables

**Table 1:** The clinical characteristics of PDTC and WDTC patients.

		Poorly differentiated carcinoma	Well-differentiated papillary carcinoma	*P* value
Gender (M/F)		7/22	44/183	n.s.

Age (median; range)		57 (20–83)	54 (11–77)	n.s.

Tumor size (cm)		3.171.8	1.9771.3	*P* < .0001

Ex*1	0	13	129	n.s., *P* = .078

	1	7	65	
	2	9	33	

Lymph node involvement*2	0	8	73	n.s.

	1a	15	82	
	1b	6	68	

Pathological subtypes	Solid	8	—	—
	Insular	5		
	Trabecular	7		
	Solid & trabecular	9		

Recurrent sites	Lymph node	7	13	—

	Lung	2	6	
	Bone	0	1	
	Others	2	2	

Median followup Period (months)		75.9	89.3	n.s.

*1 EX1: infiltrating to adjacent muscle and/or fat tissue, EX2: infiltrating to adjacent organs such as trachea, esophagus, and so forth.

*2 1a: central cervical lymph node involvement, 1b: lateral cervical lymphnode involvement.

**Table 2:** The clinical characteristics of patients with focal and diffuse component of PDTC.

		Focal component	Diffuse component	*P* value
Number		15	14	—

Gender (M/F)		4/15	7/14	n.s.

Age (median; range)		57 (20–71)	58 (22–83)	n.s.

Tumor size (cm)		2.7571.4	3.3972.1	n.s.

Ex*1	0	9	4	*P* < .01

	1	5	2	
	2	1	8	

Lymph node involvement*2	0	5	3	n.s.

	1a	8	7	
	1b	2	4	

Pathological subtypes	Solid	4	3	n.s.
	Insular	2	3	
	Trabecular	4	2	
	Solid & trabecular	5	4	

Local recurrence	P	4	5	n.s.
	N	11	9	

Distant recurrence	P	1	2	n.s.
	N	14	12	

P: positive, N: negative

*1 EX1: infiltrating to adjacent muscle and/or fat tissue, EX2: infiltrating to adjacent organs such as trachea, esophagus, and so forth.

*2 1a: central cervical lymph node involvement, 1b: lateral cervical lymphnode involvement.

**Table:** (a) For DSF

Factors	Relative risk	C.I.	*P* value
Poor differentiation	4.456	1.953–10.167	*P* < .0005
Extrathyroidal infiltration	2.898	1.278–6.572	*P* < .05

**Table:** (b) For cause-specific survival

Factors	Relative risk	C.I.	*P* value
Poor differentiation	9.343	1.314–66.453	*P* < .05
Older age	1.306	1.103–1.547	*P* < .005

C.I.: confidence interval
